# Reduced projection angles for binary tomography with particle aggregation

**DOI:** 10.1007/s12065-016-0140-7

**Published:** 2016-08-08

**Authors:** Mohammad Majid al-Rifaie, Tim Blackwell

**Affiliations:** Department of Computing, Goldsmiths, University of London, London, SE14 6NW UK

**Keywords:** Binary tomography, Discrete tomography, Particle aggregation, Underdetermined linear systems, Reduced projections

## Abstract

This paper extends particle aggregate reconstruction technique (PART), a reconstruction algorithm for binary tomography based on the movement of particles. PART supposes that pixel values are particles, and that particles diffuse through the image, staying together in regions of uniform pixel value known as aggregates. In this work, a variation of this algorithm is proposed and a focus is placed on reducing the number of projections and whether this impacts the reconstruction of images. The algorithm is tested on three phantoms of varying sizes and numbers of forward projections and compared to filtered back projection, a random search algorithm and to SART, a standard algebraic reconstruction method. It is shown that the proposed algorithm outperforms the aforementioned algorithms on small numbers of projections. This potentially makes the algorithm attractive in scenarios where collecting less projection data are inevitable.

## Introduction

Tomographic reconstruction is the process of inferring the internal structure of an object from a set of projected images. The projected images are records of the quantity of penetrating radiation that has passed through, or has been emitted from the interior of, the object in question. There are many applications, ranging from medical imaging (CT, SPECT, PET and MRI) [4, 5, 18] to oceanography (seismic tomography) [16] and quantum tomography (quantum state tomography) [6].

Although an exact reconstruction is possible by use of the inverse Radon transform, in practice the discrete nature of the imaging, and the finite number of available projections, mean that approximate and discrete techniques must be employed. The continuous density distribution of the object is modelled as a grid of pixels and the projections are acquired in bins because cameras consist of arrays of detectors of finite size [5].

Even after discrete modelling, the remaining mathematical problem may be ill-defined due to underdetermination: the number of independent relationships amongst the unknown quantities is fewer than their number. As a result, the solution of the inverse problem is not unique, and indeed very many solutions might exist.

This incompleteness of data arises from cost, time and geometrical concerns. For instance, the importance of cost reduction in industrial applications results in shortened scan duration and fewer projected images; similarly, in electron tomography, the damage caused to the sample by the electron beam reduces the number of collectable projections [15].

The classical filtered back projection [8, 13] technique is a relatively quick and effective reconstruction procedure. However, increasing computation power means that algebraic reconstruction techniques (algebraic-RT or ART) are gaining prominence. This is due to ART’s potential for greater accuracy, albeit at increased time of execution.

The first ART algorithm was a rediscovery [7] of the Kaczmarz method for solving linear equations [12]. An improved Kaczmarz method for image reconstruction, SART, (simultaneous-ART) was proposed by Andersen and Kac [2]. SART remains popular to this day and has been the subject of mathematical analysis (for example, [10]).

Prior knowledge can inform algorithms and speed up computation. For example, if it is known that the object is composed of just a few regions of homogeneous density, discrete tomography can be employed. The aim is to reconstruct an image that is composed of just a few greyscale values. And, as an extreme instance of discrete tomography, if just two greyscale values are assumed, corresponding to the interior and exterior of the object, the problem is to find a binary reconstruction [9].

The aim of this paper is to further investigate a binary reconstruction technique [1] based on the aggregation of particles. The idea is to suppose that pixel values 0 and 1 represent particles that may be absent or present in a particular cell (a pixel), and for particles to move freely until they meet, and thereupon “stick” to, clusters of other particles, subject to a concomitant reduction in error. The underlying assumption is that the preferred solutions to the inverse problem will be those solutions that are more homogeneous. Particles will, therefore, tend to move to unoccupied pixels with a greater neighbourhood count. The selection of a particle for movement was random in the previous version of PART [1]; this meant that many moves had to be rejected. In the updated version of reported here, isolated particles are preferentially selected for movement.

The paper continues with an overview of tomography and of reconstruction. Then, the aggregation algorithm, Particle aggregate-RT (PART) is specified along with its updated version; after highlighting the importance of smaller number of projections, a section detailing a sequence of experiments compares the performance of the updated version (referred to as PART 2), to the original PART algorithm, PART 1, SART, random search (RS) and filtered back projection (FBP) on a number of phantoms (i.e. pre-prepared exact images). Additionally, in a second set of experiments, the newly proposed algorithm, is analysed under several number of projections and is compared against the other algorithms. The paper ends with a summary of the main findings and suggestions for future research.

## Tomography and algebraic reconstruction

**Fig. 1:**
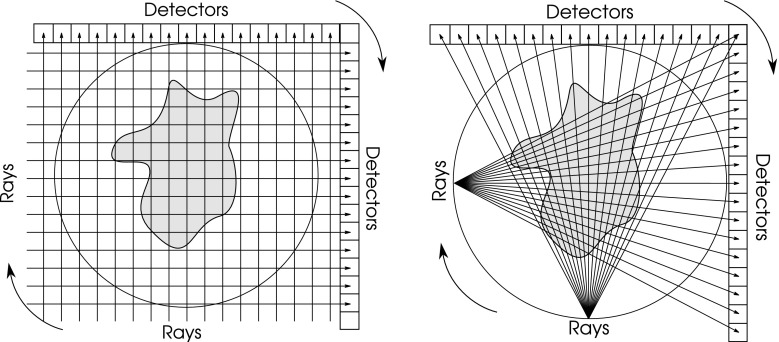
Tomography geometry. *Left* parallel beam geometry; *right* fan beam geometry

There are two important imaging modalities, parallel beam and fan beam tomography. In either modality, an array of detectors is rotated to lie at a number of (usually) equally spaced angles in $[0, \pi )$. Figure 1 shows the two modalities and the pixellated representation of the object. Ideally, if the detectors have perfect collimators, each detector will record the amount of radiation received in a finite width beam.

However, an approximate model of the physical measurement must be built in order to formalise the mathematical reconstruction problem. This approximation is called the forward model. Beams are typically modelled by parallel rays (Fig. 1-left). Each ray is incident on the centre of each detector or projection bin. The imaging process is approximated by a projection matrix $A \in \mathbb {R}_{\ge 0}^{m \times n }$ where *m* is the total number of rays collected (equal to the number of rays at each projection angle multiplied by the number of projection angles) and *n* is the number of pixels in the reconstructed image. If $b \in \mathbb {R}^m$ is a vector of detector values, the continuous/discrete reconstruction problem can be stated as:

1 $$\begin{aligned} \text {find } x {\left\{ \begin{array}{ll} \in \mathbb {R}^n \\ \in \{0, 1, \ldots , k-1\}^n, k > 1 \\ \end{array}\right. } \text { such that }\,\, Ax = b. \end{aligned}$$

The binary problem is $k = 2$ i.e. with $x \in \{0, 1\}^n$.

**Fig. 2:**
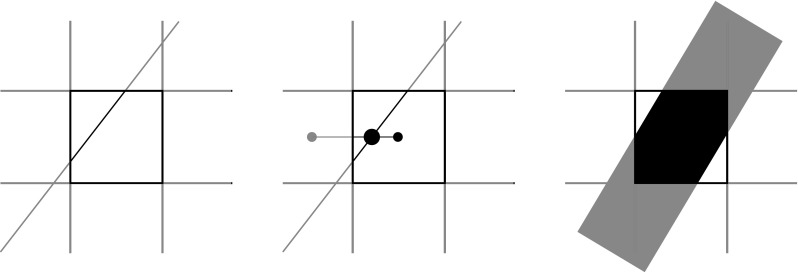
Three projection models. From *left to right*: *line*, Joseph and strip models

The methods used to computed the intersection between the ray and the pixels vary. One such common method is the *line model*, where the entries in the projection matrix, $a_{ij}$, are computed by measuring the length of the intersection between the line of the ray and the pixel (see Fig. 2-left). In this model, when the projection rays are parallel to the horizontal or vertical axes, the weight function exhibits two discontinuities; these, caused by floating point error, could lead to wrongly setting the weight entries to 0 instead of 1, or vice versa.

In order to overcome this issue, Joseph’s weighting scheme [11] could be used instead. In this model, the interpolation coefficients are calculated when following the line column by column or row by row (based on the projection angle chosen). Thus, linear interpolation between the centres of the two adjacent pixels are applied. See Fig. 2-middle.

In another model, the strip mode, strips are used with width larger than a unit instead of lines. Therefore, the intersection area between strip *i* and pixel *j* determines the weight $a_{ij}$ as displayed in Fig. 2-right. While in the the strip model the column sums of the projection matrix is constant, this is does not hold for the line and Joseph models.

In this work, in order to compute the entries $A_{ij}$ of the projection matrix, a more refined *line model* which uses the length of the intersection between the ray and the pixel is used.

Since the equation $Ax = b$ is, in general, underdetermined, it cannot be inverted. Instead an approximate solution *y* must be obtained (for example, by FBP, or SART). This trial solution is forward projected according to the measurement model:

$$\begin{aligned} Ay&=c \end{aligned}$$

with an associated $l_p$ projection error

$$\begin{aligned} \epsilon (y)&= || b - c ||_p \end{aligned}$$

where the $l_p, p \ge 1$, norm is defined

$$\begin{aligned} || v ||_p \equiv \left( \sum |x|^p \right) ^{\frac{1}{p}}. \end{aligned}$$

An iterative scheme will produce a sequence of candidate solutions, $y^{(k)}, k=1,2,\ldots$, of decreasing error.

A zero projection error might yield a reconstructed solution *y* that is not identical to the original object *x*. This is due to underdetermination. However, in cases where the reference image is known, the proximity of *y* to *x* offers a second and more stringent measure of algorithm performance.

Consider the following measures:

2 $$e_1= || b - c ||_1$$

3 $$e_2= || y - x ||_1$$

A zero value of $e_1$ solves the problem $Ay = b$ but does not guarantee reconstruction proximity. $e_2$ provides a check: a value of zero corresponds to a reconstructed image that is the exact replica of the original.

## Reconstruction by particle aggregation

In many applications, the reconstructed image is expected to consist of patches of various sizes of uniform pixel value, since many physical objects of interest consist of uniform structures. Non-uniform regions with randomly varying pixel values would be construed as noisy and unphysical. Relevant reconstructed images are therefore those with low entropy.

This observation suggests the following assumption: given a number of distinct candidate reconstructions, $\{y : Ay = c \}$, with identical error $\epsilon (y)$, the preferred reconstruction is the one with the lowest entropy (or one of the reconstructions of lowest entropy, in the case of non-uniqueness). It would clearly be beneficial to equip a reconstruction algorithm with this assumption, under those conditions where the assumption might be expected to hold.

The principal idea underlying the aggregation technique proposed in this paper and motivated by the low-entropy assumption, is to suppose that pixel values are mobile particles, moving from pixel to pixel. The low-entropy assumption is implemented by requiring that particles stick together in clusters to form aggregates of uniform pixel value.

A model of aggregation for any random deposition process that is dominated by diffusive transport, for example electodeposition and mineral growth, was proposed by Witten and Sander [20]. Their model, known as Diffusion Limited Aggregation or DLA, is remarkably simple: a particle is released from a random point on a boundary and subsequently follows a random walk until it strikes a stationary particle at some location within the enclosing boundary. The walking particle sticks to the stationary particle and another particle is released. Surprisingly complex dendrite-like clusters with fractal structure are formed by repeated application of this simple rule.

The reconstruction problem is converted into particle aggregation with the following correspondence:

- image $x \rightarrow$ configuration of particles,
- pixels $$\rightarrow$$ cells
- pixel values 1/0 $\rightarrow$ presence/absence of a single particle,
- image $\rightarrow$ a grid of cells.

Furthermore, an objective function

- error $\rightarrow$ objective function

converts the growth model into an *optimisation* problem: only those aggregates that lower the objective function are permitted to form.

A direct implementation of DLA as a reconstructive process would be very expensive since a randomly walking particle might pass by many isolated cells before arriving at a boundary cell; diffusion can be accelerated by causing a particle to jump from cell *a* to a vacant cell *b*, picked uniformly at random from all vacant cells. Although a jump has been made, the particle might not necessarily ‘stick’.

Suppose a particle has jumped from *a* to *b* and that *b* is a boundary cell of a particle cluster^1^. We might suppose that whether the particle sticks or not to the cluster is conditional on the number of occupied neighbours of the boundary cell *b* relative to the neighbour count for cell *a* - with higher neighbourhood counts being preferred, and on the fitness of the new configuration. There are a number of ways to deal with a particle that has jumped to a vacant cell but does not stick. For example, it could simply return to *a*.

With these considerations in mind, the particle aggregate reconstruction technique (PART) can be specified.^2^ Algorithm 1 specifies an application of PART to a single particle. Here, *y* is the reconstructed image, Select (see Algorithm 2) returns pixels $a, b \in y, a \ne b,$ such that *a* is occupied and *b* is empty. *n* is the number of occupied cells in the neighbourhood (Moore or von Neumann) of a particular cell and $\epsilon (a \rightarrow b)$ is the error of the new image with the pixel *a* set to zero and pixel *b* set to 1. *u* is a sample drawn from *U*(0, 1) (the uniform distribution on [0, 1]).

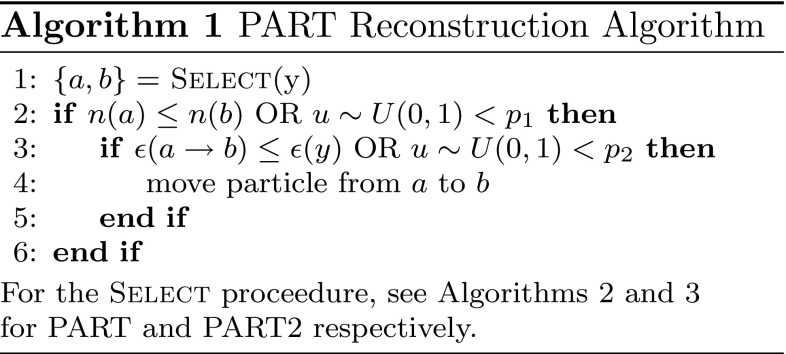



The algorithm has two parameters $p_1$ and $p_2$. $p_1$ governs the influence of the local neighbourhood constraint: the requirement to move to a neighbourhood of higher local particle density. $p_1 = 1$ corresponds to a random search and the neighbourhood constraint is ignored. A move $a \rightarrow b$ will always be attempted even if the neighbourhood function *n* is lowered.

In contrast, $p_2$ governs the influence of the global constraint on the particle configuration as a whole. If $p_2 = 0$, a move $a \rightarrow b$ will always be rejected if it does not lower or equal the current error. The algorithm is greedy. If $p_2 > 0$, the algorithm is not greedy and a configuration with higher error will be accepted with probability $p_2$. Movement away from a local minima of $\epsilon$ can occur. In principle, $p_2$ might depend on the change in error (and on a steadily reducing temperature parameter as in simulated annealing). While finding the optimal value for $p_2$ is not explored in this paper (and $p_2$ is set to zero here), optimising this parameter is a subject of an ongoing research.

Algorithm 1 specifies a trial update of a single particle. Each application incurs a cost of a single function evaluation ($\epsilon (y)$). The algorithm is iterated until zero error or until a set number of function evaluations (FEs) has been achieved.

As stated by Reynolds [17], the three simple rules of interaction in flocks are collision avoidance, velocity matching and flock centring. Swarms differ from flocks in the sense that there is no velocity matching. The aggregating particles in PART can be considered as individuals in a swarm. The dynamic rules of particles swarms are of the form:

1. **If** too close or colliding to neighbouring particles, move away
2. **Else if** too far from neighbours, move closer.

where rule 1 opposes crowding and rule 2 brings the particles together in a swarm. The single occupancy condition implements the anti-crowding rule, and the (conditional) move to a neighbourhood of higher particle density, as measured by the neighbourhood function *n* implements rule 2. The error function $\epsilon (y)$ imposes a global constraint on the swarm as a whole.

In an altered version of PART, further emphasis is placed on the aggregation of particles by more systematically choosing *isolated* particles as more suitable *a* pixels to be placed in *b* pixels. This is arranged by creating an ordered list of particles’ neighbourhood counts (see Algorithms 1 and 3).

## Experiments and results

In [1], three experiments were conducted in order to investigate the performance of PART 1 in the context of binary image reconstruction: the first and preliminary, experiment, aimed at finding a suitable value for the local constraint parameter $p_1$ for a single phantom of one size only. This value is set to $p_1 = 0.1$; the second experiment investigated the convergence properties of PART 1 and random search, which can be seen as a limiting case of PART 1. The results demonstrate the outperformance of PART 1 in all cases except when reference images (or phantoms) are only noise, in which case, as expected, random search performs better. The final experiment provided a comparison between random search, the commonly used reconstruction algorithm, simultaneous algebraic reconstruction technique (SART), and PART 1 with $p_1$ set to the empirical value determined in the first experiment. The result of this set of experiments demonstrated that PART 1 converges rapidly when compared to random search for phantoms with all nonzero pixel values occurring in connected regions. And in the case that there are isolated nonzero values pixels, PART 1 will find better reconstructions at fewer iterations. Additionally, PART 1 performs (statistically) significantly well when compared to random search and a standard algebraic reconstruction technique for $32 \times 32$ and $64 \times 64$ phantoms, except for the case of isolated nonzero pixel values; it is also shown that for a larger $128 \times 128$ phantom with proportionally fewer angles of projection, PART wins out over random search and SART.

In this work further experiments are conducted with the focus on the important issue of reconstruction with fewer number of projections, as in practice, merely a small number of projections can be collected, thus giving rise to what is known as *limited data problems*. There are several reasons behind this, including cost, time, and geometrical constraints. For instance, the importance of cost reduction in the industry applications results in shortened scan duration, which in turn leads to less projections; similarly, in electron tomography, the damage caused to the sample by the electron beam reduces the number of collectable projections [15]; and in nuclear imaging, reducing the number of projection means reducing the duration in which patients should be exposed to radioactive materials as well as the inconvenience of long scanning time.

This stresses that algorithms need to return sufficiently suitable approximations of the original phantoms even with smaller number of projections, which is what some of the experiments in this section are allocated to. In this section, PART 2 is contrasted against PART 1, RS, SART and FBP over all the phantoms used in this work.

### Methodology

#### Forward model

The acquisition geometry used for the experiments is parallel beam topology and the experiments use simulated objects (i.e. virtual phantoms). In all cases, the elements of the projection matrix were calculated from the line model.

#### Phantoms

**Fig. 3:**
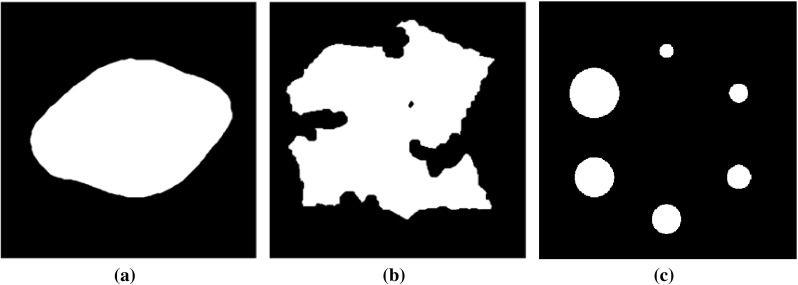
Phantom images used in the experiments. **a** Phantom 1, **b** phantom 2, **c** phantom 3

Phantoms 1 and 2 (see Fig. 3) are commonly used in binary tomography [19] and the third phantom resembles the Jaszczak phantom used to calibrate the SPECT and PET scanning machines. The size of all the phantoms is 512 $\times$ 512. To carry out the experiments in images with different sizes, the phantoms or reference images have been scaled to create images of varying sizes (namely, $64 \times 64$ and $128 \times 128$).

#### PART 1 & 2

PART is used with the Moore neighbourhood. There are a number of alternatives for line 1 of Algorithm 1, the selection step in PART. The purpose of this step is to find an occupied cell, *a*, and a vacant cell, *b*. The following experiments use random selection: *a* and *b* are selected uniformly at random from the sets of all occupied/unoccupied cells. A list implementation would have been efficient, but since the numbers of occupied/unoccupied cells is roughly similar, uniform sampling over the entire grid *y* was used due to the ease of implementation and small time overhead. Algorithms 2 and 3 specify Select for PART 1 & 2; *U*(*y*) is a uniform random selection of a single cell from the grid *y*. The value of the global constraint parameter $p_2$ was fixed, in all experiments, to zero.

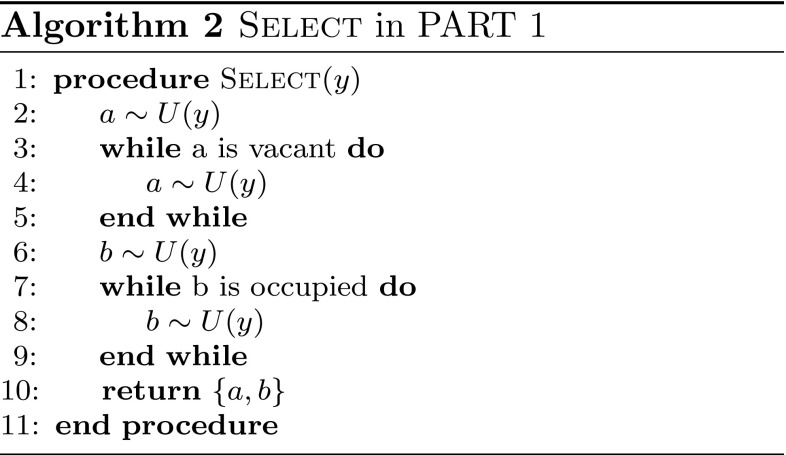

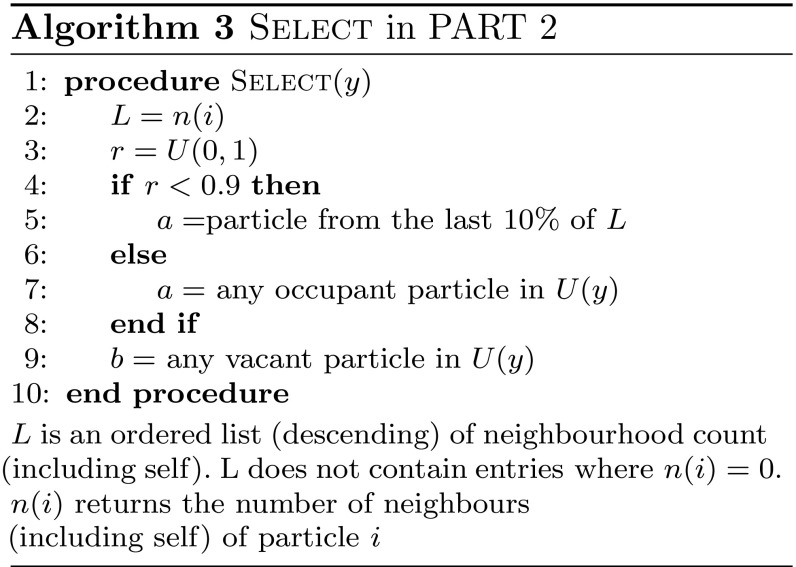





#### Random search (RS)

For the purposes of these experiments, random search is defined as the PART algorithm with the neighbourhood parameter $p_1$ set to 1 with the consequence that a particle will always attempt a move to an unoccupied cell *b* even if the neighbour count of *b*, *n*(*b*), is less than *n*(*a*).

#### Simultaneous algebraic reconstruction technique (SART)

The implementation of SART used here was based on Andersen and Kac’s algorithm, [2]. The projection angles were selected uniformly at random [3]. The value of the relaxation parameter $\lambda$ was set to 1.9 in accordance with the recommendation of [14].

SART needs to be modified for binary reconstruction since in the unaltered form SART produces a continuum of pixel values. The following modifications were made: any negative pixel values occurring after updating at any angle were set to zero; the final image *y* after updating all projection angles was normalised so that the total pixel value count of the phantom image and the reconstructed image were equal; *y* was thresholded at the average pixel value so that values below the average were set to zero, values above or equal tot he average were set to 1.

#### Filtered back projection (FBP)

In order to provide a more comprehensive account to the experiments conducted in this work, FBP algorithm is also used. FBP algorithm is capable of fast and adequate reconstruction, but requires a large number of projections. FBP generates an image in a single iteration.

#### Measure

The principle performance measure is the image proximity $e_2 = ||y - x||_1$ (defined in Eq. 3) where, for the phantom image $x, Ax = b$ and for the reconstructed image, $Ay = c$. However, while the algorithm uses the projection error $e_1 = || b- c ||_1$ as the objective function. This is because, in practice, *x* is unknown.

### Experiments and results

In this section, phantoms of $64 \times 64$ and $128 \times 128$ are used with 8 and 5 projections respectively. In these experiments $p_1 = 0.10$, $p_2 = 0.00$ and 30 runs were conducted for test in order to acquire adequate statistics. The termination condition for each run is 20, 000 function evaluations (FEs). For the purposes of this study, the number of FEs does not vary with the size of the phantoms and the number of projections.

In this section the five algorithms are used (e.g. PART 1 & 2, RS, SART and FBP) on Phantoms I, II, and III (see Fig. 3).

**Fig. 4:**
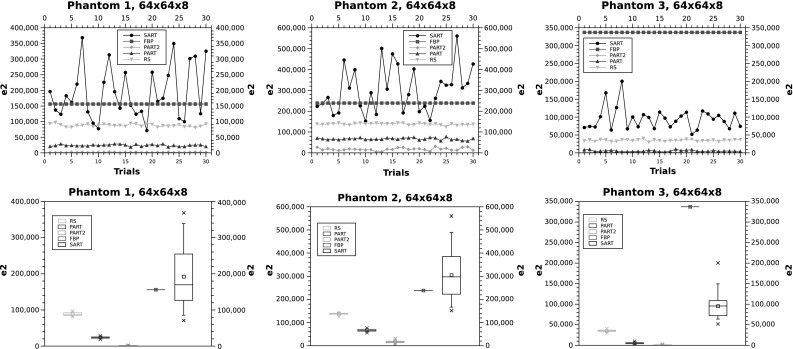
$$e_2$$ in $$64 \times 64 \times 8$$

The results of running the five algorithms on the three phantoms in $64 \times 64 \times 8$ are shown in Fig. 4. The results show a clear and almost homogeneous picture on the performance of the algorithms. The algorithms’ performance ranking appear in the following order: PART 2, PART 1, RS, FBP and SART. The only exception appears in phantom 3 where SART outperforms FBP.

**Fig. 5:**
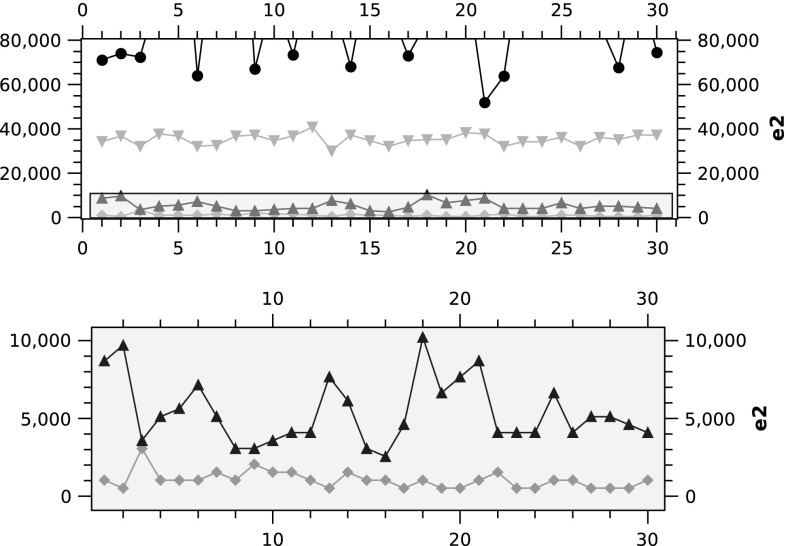
$e_2$ in $64 \times 64 \times 8$ for phantom 3. In the *bottom plot*, PART 2 is shown in *green* and PART 1 is highlighted in *blue* (color figure online)

Given the large error margin of FBP and SART, Fig. 4 does not clearly show the difference between PART 1 & 2, where Fig. 5 zooms into the graph to show the difference between these two variations of PART.

**Fig. 6:**
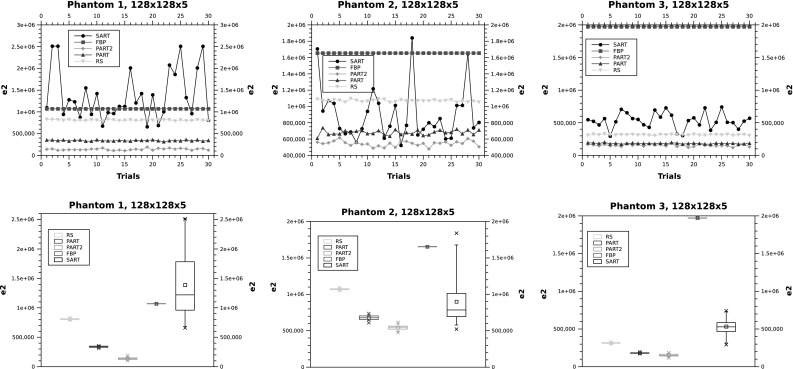
$$e_2$$ in $$128 \times 128 \times 5$$

The results of running the five algorithms on the three phantoms in $128 \times 128 \times 5$ are shown in Fig. 6. The results match the previous observations on the smaller phantoms, with the difference that SART outperforms FBP in phantoms 2 and 3. The performance ranking of the other algorithms is maintained (i.e. PART 2, PART 1 and then RS).

**Fig. 7:**
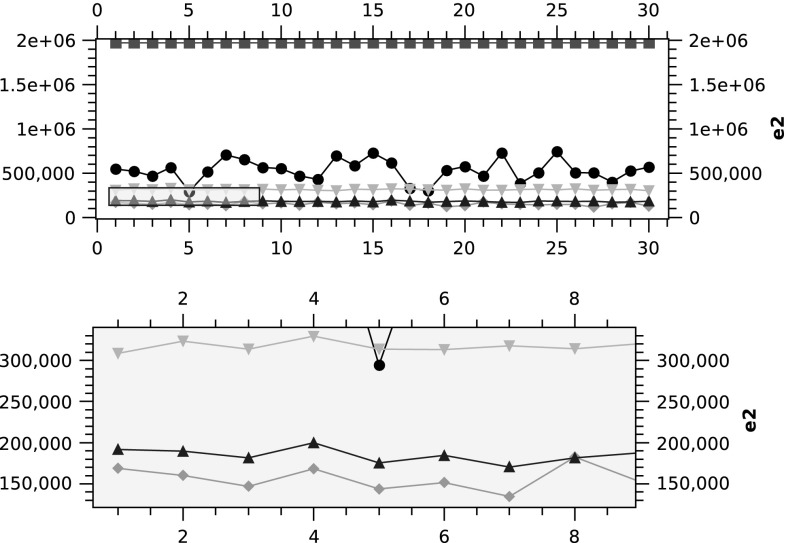
$e_2$ in $128 \times 128 \times 5$ for Phantom 3. In the *bottom plot*, PART 2 is shown in *green* and PART 1 is highlighted in *blue* (color figure online)

Again, in order to visually compare the results of PART algorithms in phantom 3, Fig. 7 illustrates the difference on a few of the experiments, zooming into the graph.

The summary of figures from which the plots are derived are reported in Table 1. This table shows the performance of the five algorithms used in this work when reconstructing three phantoms in $64 \times 64 \times 8$ and $128 \times 128 \times 5$ configurations.

**Table 1:** Comparing PART2, PART1, RS, SART and FBP in $64 \times 64 \times 8$ and $128 \times 128 \times 5$ experiments

	Min	Max	Median	Mean	StDev
(a) $$64 \times 64 \times 8$$					
Phantom 1					
PART2	510	3060	1530	1445	600.21
PART1	18,360	28,560	23,460	23,749	2966.79
RS	81,090	97,410	87,975	88,281	4263.5
SART	71,145	368,475	169,575	192,508	85,173.71
FBP	156,190	156,190	156,190	156,190	0
Phantom 2					
PART2	5100	31,620	16,320	17,493	6556.06
PART1	56,610	77,010	66,555	66,759	4932.56
RS	124,950	143,820	139,230	138,380	4216.93
SART	151,725	560,490	297,585	305,209.5	109,065.36
FBP	238,960	238,960	238,960	238,960	0
Phantom 3					
PART2	510	3060	1020	1037	560.01
PART1	2550	10,200	4845	5389	2073.51
RS	30,090	40,800	35,190	35,275	2384.91
SART	52,020	200,430	95,880	95,557	31,399.4
FBP	337,340	337,340	337,340	337,340	0
(b) $$128 \times 128 \times 5$$					
Phantom 1					
PART2	107,610	191,760	135,405	138,669	20,283.75
PART1	313,650	357,510	339,150	339,745	11,298.13
RS	786,930	834,360	809,370	810,662	11,662.70
SART	659,175	2,512,260	1,219,410	1,389,206	588,415.16
FBP	1,070,800	1,070,800	1,070,800	1,070,800	0.00
Phantom 2					
PART2	477,870	616,590	548,760	544,561	32,452.80
PART1	609,450	736,950	676,515	678,504	29,424.83
RS	1,049,580	1,095,480	1,069,725	1,070,286	12,500.01
SART	522,750	1,840,845	785,017.5	901,340	330,794.76
FBP	1,654,200	1,654,200	1,654,200	1,654,200	0.00
Phantom 3					
PART2	116,280	185,640	151,725	152,507	17,061.81
PART1	170,340	199,920	182,070	182,121	7035.41
RS	302,430	329,460	314,415	315,112	6456.23
SART	294,270	742,305	528,615	533,103	120,685.03
FBP	1,971,500	1,971,500	1,971,500	1,971,500	0.00

**Table 2:** Statistical analysis of the performance of the algorithms

	PART2–PART1	PART2–RS	PART2–SART	PART2–FBP
(a) $$64 \times 64 \times 8$$				
Phantom 1	X–o	X–o	X–o	X–o
Phantom 2	X–o	X–o	X–o	X–o
Phantom 3	X–o	X–o	X–o	X–o
$\sum$	3–0	3–0	3–0	3–0
(b) $$128 \times 128 \times 5$$				
Phantom 1	X–o	X–o	X–o	X–o
Phantom 2	X–o	X–o	X–o	X–o
Phantom 3	X–o	X–o	X–o	X–o
$\sum$	3–0	3–0	3–0	3–0

Based on Wilcoxon $1\times1$ Non-Parametric Statistical Test, if the *error* difference between each pair of algorithms is significant at the 5 % level, the pairs are marked. X–o shows that the left algorithm is significantly outperforming its counterpart algorithm; and o–X shows that the right algorithm is significantly better than the one on the left. The figures, n – m, in the last row present a count of the number of X’s and o’s in the respective columns

For the purpose of providing a more meaningful analysis and comparison of results, a statistical analysis would help identify the presence of any significant difference in the behaviour of the algorithms (i.e. finding $e_2$) across the phantoms. For this reason, a Wilcoxon $1\times 1$ non-parametric statistical test is deployed.

Investigating Table 2 validates the previous finding and confirms that PART 2 exhibit a *statistically significant* difference when compared to the rest of the algorithms used in this study. This result holds both for the experiments conducted on phantoms of size $64 \times 64$ with 8 projection angles and phantoms of size $128 \times 128$ with 5 projection angles.

**Fig. 8:**
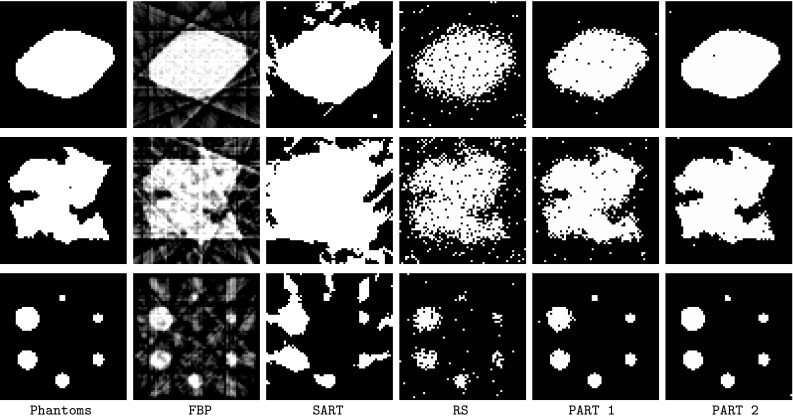
Reconstructed phantoms in $64 \times 64 \times 8$. From *left to right* original phantoms, FBP, SART, RS, PART1, PART2

**Fig. 9:**
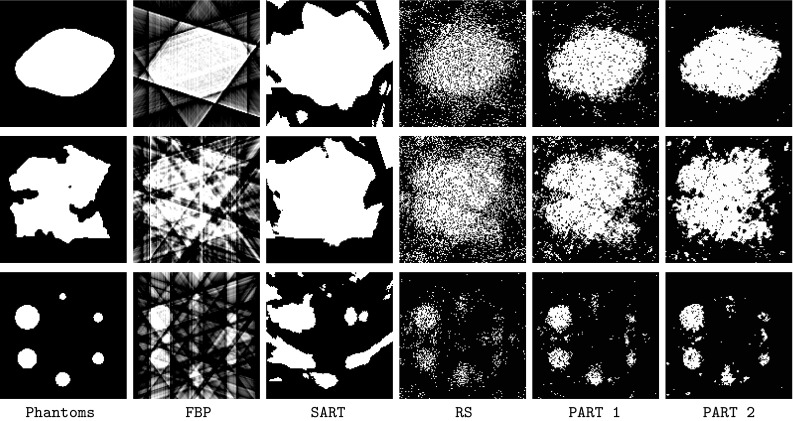
Reconstructed phantoms in $128 \times 128 \times 5$. From *left to right* original phantoms, FBP, SART, RS, PART1, PART2

Figures 8 and 9 present the reconstructed phantoms by FBP, SART, RS, PART1 and PART2 using two configurations $64 \times 64 \times 8$ and $128 \times 128 \times 5$.

These results indicate that the proposed algorithm performs better than the rest of the algorithms when a small number of projection angles are deployed. In contrast to the previous experiments reported in [1], where a larger number of projections where used (i.e. $\alpha = \sqrt{n} / 2$, where $\alpha$ is the number of projection angles, and *n* is the number of pixels in the phantom), in these experiments a small number of projections where deployed, therefore adding to the undeterministic nature and thus complexity of the problem. Despite this, the proposed algorithm is exhibiting a competitive performance. To verify the strength of PART and explore the reduction in the number of projections, a set of experiments are designed and the results are reported in the next section.

### Impact of the number of projection angles

In this section one phantom is used with the image size of $64 \times 64$. The aim of this experiment is to investigate the role of the number of projection angles on the performance of the reconstructing algorithms. The three algorithms picked are PART 2 along with the classically used SART and FBP algorithms. Both SART and FBP are not able to improve their reconstructed images after the end of the iteration. However, PART in principle, can iteratively reconstruct the phantoms. In this experiment each algorithm is run 10 times and the termination criteria for PART 2 is reaching 50,000 FEs.

**Fig. 10:**
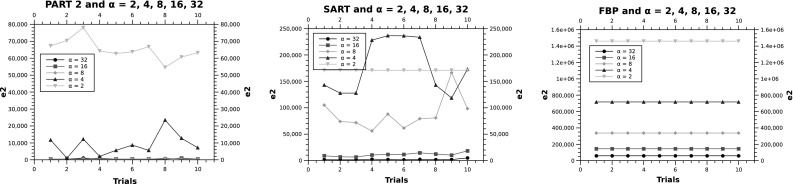
Varying number of projection angles ($\alpha$) for PART2, SART and FBP

The plots in Fig. 10 clearly illustrate the impact of the number of projections on the quality of the reconstructed images (measured using $e_2$). In this set of experiments, FBP shows a clear impact of $\alpha$ on its performance (i.e. the higher the number of projections, the smaller the error); this picture changes slightly in SART where for example, in several runs, the error in $\alpha =2$ is better than $\alpha = 4$. Consistent with the previous experiments where PART 2 is outperforming the other algorithms, the algorithm still maintains its superiority in terms of the resultant $e_2$. Table 3 presents the summary of the numerical values of these experiments.

**Table 3:** Varying values of $\alpha$ for PART2, SART and FBP in $64 \times 64$

	Min	Max	Median	Mean	StDev
PART 2					
$\alpha =2$	54,570	78,030	64,005	65,178	6189.49
$\alpha =4$	1020	23,460	7905	9027	6495.91
$\alpha =8$	510	1020	510	561	161.28
$\alpha =16$	510	1020	510	612	215.03
$\alpha =32$	510	1020	510	612	215.03
SART					
$\alpha =2$	171,105	171,105	171,105	171,105	0.00
$\alpha =4$	118,575	236,640	158,100	176,817	51,089.77
$\alpha =8$	55,845	166,515	79,943	88,077	31,420.86
$\alpha =16$	6630	18,360	10,965	11,118	3492.66
$\alpha =32$	1275	4845	1658	2015	1084.87
FBP					
$\alpha =2$	1,462,900	1,462,900	1,462,900	1,462,900	0.00
$\alpha =4$	717,020	717,020	717,020	717,020	0.00
$\alpha =8$	337,340	337,340	337,340	337,340	0.00
$\alpha =16$	144,530	144,530	144,530	144,530	0.00
$\alpha =32$	59,328	59,328	59,328	59,328	0.00

**Fig. 11:**
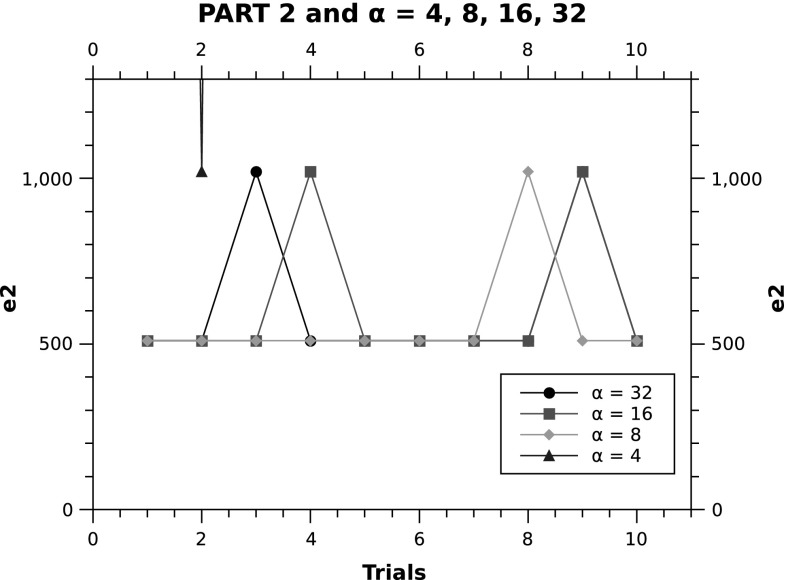
PART2: Varying number of projection angles ($\alpha$)

One of the interesting observations in the PART 2 plot is the presence of some instances where $\alpha = 4$ finds equally good error values in comparison with $\alpha =8,16,32$. This suggests that PART 2 is less dependant on the value of $\alpha$ and can perform well even in cases where only smaller number of projections can be obtained in the real-world experiments and clinical setups. Figure 11 shows a closer view on PART 2 with $\alpha =4,8,16,32$. The error of 510 shared by most trials with $\alpha =8,16,32$ means that only two pixels are misplaced (i.e. two white pixels, $510 = 2 \times 255$).

## Conclusions

This paper extends the previously introduced particle aggregate reconstruction technique (PART) with emphasised focus on the aggregation of isolated particles. PART is based on the idea that an image can be interpreted as a grid of cells populated by particles. Pixel values represent cell occupancy; particles are mobile and diffuse throughout the grid by random jumps, preferably landing adjacent to regions of increased particle density. The algorithm is intuitive, and easily implemented.

This work also puts a particular emphasis on the reduction in the projection angles and therefore aiming to use less data to reconstruct the phantom images.

A number of experiments were designed based on three phantoms in two sizes of $64 \times 64$ and $128 \times 128$ and the proposed variation of PART algorithm is contrasted against FBP, simultaneous algebraic reconstruction technique (SART) as well as random search (RS).

Based on the results and in terms of the error, the dominance of PART over the aforementioned techniques is suggestive. Furthermore, the results demonstrate the *statistically significant* outperformance of PART in all instances.

An experiment was designed to show the impact of the number of projections on the reconstruction quality of the phantom images. It is shown that PART is less sensitive to the number of projection angles, making the algorithm attractive when less data are available, or in situations where collecting less projection data are inevitable (i.e. in medical scenarios where patients cannot be kept for long duration for the scanning purposes, or where long exposure to radiation is lethal).

One of the main research questions is whether aggregation by particle diffusion can be extended to the general discrete case, which is the topic of ongoing research.

## Footnotes

1 Note that the boundary might lie within the cluster i.e. bounding a hole.

2 PART source code can be downloaded from http://doc.gold.ac.uk/~map01mm/PART/.
